# Editorial: Disabled people's health

**DOI:** 10.3389/fpubh.2024.1485424

**Published:** 2024-09-18

**Authors:** Saengryeol Park, So-Youn Park, Minha Hong, Ahmad M. Osailan, In-Hwan Oh

**Affiliations:** ^1^Department of Physical Education, College of Education, Chonnam National University, Gwangju, Republic of Korea; ^2^Department of Medical Education and Humanities, Kyung Hee University College of Medicine, Seoul, Republic of Korea; ^3^Department of Psychiatry, Myongji Hospital, Hanyang University College of Medicine, Seoul, Republic of Korea; ^4^Department of Health and Rehabilitation Sciences, College of Applied Medical Sciences, Prince Sattam Bin Abdulaziz University, Al-Kharj, Saudi Arabia; ^5^Department of Preventive Medicine, Kyung Hee University College of Medicine, Seoul, Republic of Korea

**Keywords:** disabilities, health status, health behavior, medical use, mental health

## 1 Introduction

Approximately 16% of the global population lives with disabilities (1). People with disabilities are at risk of developing physical and mental diseases and face health inequities due to stigma, poverty, poor education, and inaccessible healthcare systems. In addition, four-fifths of the people with disabilities come from low-to middle-income countries, and there are discrepancies in the prevalence of disabilities by age, sex, and regions (2). However, the literature is mostly limited to developed countries and healthcare providers, rather than people with disabilities themselves.

## 2 Synopsis of articles on the Research Topic

The Research Topic includes diverse content examining physical activity, mental health, toolkit development, etc., across the spectrum of this population. A total of seven articles investigated individuals, aged 0–65 years, from diverse backgrounds (people with disabilities, medical students, healthcare providers, and significant others). Two of the seven articles employed mixed methods to conduct intervention studies; three were cross-sectional studies; and there was one experimental study and one review in Thailand, United States, Cameroon, and Saudi Arabia.

### 2.1 Observational studies

Selanon and Chuangchai  examined the effect of walking distance on the subjective health of people with disabilities in 378 individuals, aged 13–65 years, covering seven types of disabilities: visual/hearing impairments, physical/mobility-related disabilities (physical group), intellectual and learning disabilities (cognitive group), and autism and emotional/behavioral disabilities (social group). Their analyses revealed that walking distance affected the subjective health of people with disabilities. The authors suggested the incorporation of environmental planning as a rehabilitation strategy to improve walking in persons with disabilities. Sayed's cross-sectional study examined hesitancy and attitudes toward COVID-19 vaccines among 186 people with disabilities, aged 30–49 years, in Saudi Arabia. Disability types included mobility disabilities, visual and hearing impairments, speech and language disorders, and mental disabilities. Participants were asked to complete a questionnaire on their previous experience, use, and trust in different sources of information and their attitudes toward the COVID-19 vaccine. This study found that Saudi Arabians with disabilities reported positive levels of COVID-19 vaccine use. Their belief in healthcare workers, social media, and TV/radio as sources of information on the COVID-19 vaccine was found to be strong. Ogle et al.'s observational study investigated 246 medical students' intolerance of uncertainty on their attitudes toward people with disabilities, and found that medical students with high levels of uncertainty intolerance showed low levels of disability attitudes.

### 2.2 Intervention studies

Widerström-Noga et al. conducted a mixed-methods study to examine the effectiveness of a pain education tool, named *SeePain*, for people with spinal cord injury, their significant others, and healthcare providers. The use of *SeePain* is 2-fold: classification of pain diagnosis and pain management. The tool was tested on 15 people, aged 30 years, with spinal cord injuries. Participants completed questionnaires on pain and qualitative interviews on content of the *SeePain*. The authors suggested that educational resources could be better delivered online in some cases. In addition, Fernandez et al. confirmed the usefulness of *SeePain* through confirmatory factor analysis. Yuh et al. developed a family centered toolkit to improve the livelihoods of people with disabilities in Cameroon, and it's effectiveness was examined in a mixed-methods study. Data were collected over 12 geographical areas in Cameroon from 26 people with disabilities aged 0–40 years via key information interviews and focus group discussions. In addition, the participants completed questionnaires four times. The results showed an improvement in the overall scores of education, health, social wellbeing, livelihood, and empowerment over 12 months. The findings indicated that the family-centered toolkit may play a role in improving the poverty and independence of people with disabilities.

### 2.3 Review

Furthermore, Mannor and Needham reviewed 41 articles, both qualitative and quantitative, on ableism to identify the social factors affecting the health of people with disabilities. In addition, the authors addressed the social definition of ableism across four social levels: institutional, internalized, structural, and interpersonal. The review revealed that the majority of the studies focused on institutional ableism, particularly in the healthcare domain, which, in turn, underestimated the representativeness of persons with disabilities. The study addressed the importance of theoretical intersectionality in ableism as a future direction.

## 3 Conclusion

This editorial investigates the impact of physical activity and toolkit-based education programs on the health of people with disabilities. Articles in this Research Topic included people from diverse backgrounds, including people with disabilities, medical students, and significant others. One finding was that physical activity plays a role in improving physical and mental health, indicating the importance of changing the physical environment of people with disabilities. In addition, educational interventions may be effective when various interested parties in the disability domain are involved in the development process. These findings have significant implications for understanding how researchers and policymakers should develop strategies to narrow the gap between policy and the real lives of people with disabilities.
